# A Rare Case of Crystal-Storing Histiocytosis of the Breast in a Patient With Undifferentiated Connective Tissue Disease

**DOI:** 10.7759/cureus.85620

**Published:** 2025-06-09

**Authors:** Mariel Bedell, Rana Naous

**Affiliations:** 1 Pathology, University of Pittsburgh Medical Center, Pittsburgh, USA

**Keywords:** breast, connective tissue disease, crystal, histiocytosis, lymphoproliferative disorder, storing

## Abstract

Crystal-storing histiocytosis (CSH) is a rare entity usually presenting microscopically as sheets of histiocytes with intralysosomal accumulation of refractile, crystalline substances. CSH is most often the result of monoclonal immunoglobulin light chain deposition in the setting of a plasma cell neoplasm or lymphoma with plasma cell differentiation. However, this condition may arise in association with autoimmune, inflammatory, infectious, and drug-related etiologies and can harbor polyclonal immunoglobulins or other crystalline materials. It usually presents in a localized fashion, with the bone marrow being the most frequent site, but may also involve multiple anatomical sites. CSH is typically identified incidentally without clinical suspicion and can be challenging to detect histologically. However, in rare cases, it may be the sole presenting sign in an undiagnosed malignancy or immune disorder. Here, we describe an unusual case of CSH, occurring as an isolated breast mass in association with an undifferentiated connective tissue disease (UCTD) with inflammatory arthritis, features of Sjögren’s (Sicca) syndrome, and subsequent laboratory findings concerning for antiphospholipid syndrome.

## Introduction

Crystal-storing histiocytosis (CSH) is a rare condition characterized by the cytoplasmic accumulation of crystalline material in histiocytic lysosomes. The crystals are most often comprised of monoclonal immunoglobulin light chains, most frequently kappa type, without specific heavy chain associations [1,2]. Involvement ranges from unifocal localized clusters of histiocytes in more than 80% of cases to generalized, multiorgan mass-like lesions [3-5]. In general, the bone marrow is nearly ubiquitously involved, but CSH may involve essentially any anatomical location, with head and neck, lymph node, lung, kidney, spleen, skin, and gastrointestinal mucosal sites predominating [3,4]. CSH is identified in the setting of an underlying lymphoproliferative or plasma cell disorder, such as multiple myeloma, lymphoplasmacytic lymphoma, monoclonal gammopathy of undetermined significance, or B-cell lymphoma in more than 75% of cases [3-6]. In the remainder of cases, non-neoplastic disorders accompany CSH, including autoimmune, inflammatory, infectious, and drug-related etiologies. In these settings, crystalline accumulation may be polyclonal or rarely even non-immunoglobulin in nature. In particular, Sjögren’s syndrome [7-10] and rheumatoid arthritis [11] are cited most often, followed by much rarer associations in inflammatory conditions such as inflammatory bowel disease and non-immunoglobulin-related etiologies of CSH, such as silicosis and clofazimine intake [12,13]. Molecular studies of CSH suggest that mutations resulting in conformational abnormalities of the immunoglobulin light chain may be key in the development of this condition [1].

This condition affects men and women at roughly equal rates and affects a broad age range, with a mean age of diagnosis around 60 years of age [3-5]. Radiologic findings of CSH are nonspecific but typically describe a well-defined, solid, nodular infiltrate with homogeneous contrast enhancement [3-5,7,14]. CSH itself is benign but may generate clinical suspicion for malignancy when mass-forming. Herein, we present a rare case of CSH incidentally discovered in the breast in association with an undifferentiated connective tissue disorder in a patient without a history of malignancy.

## Case presentation

**Figure 1:**
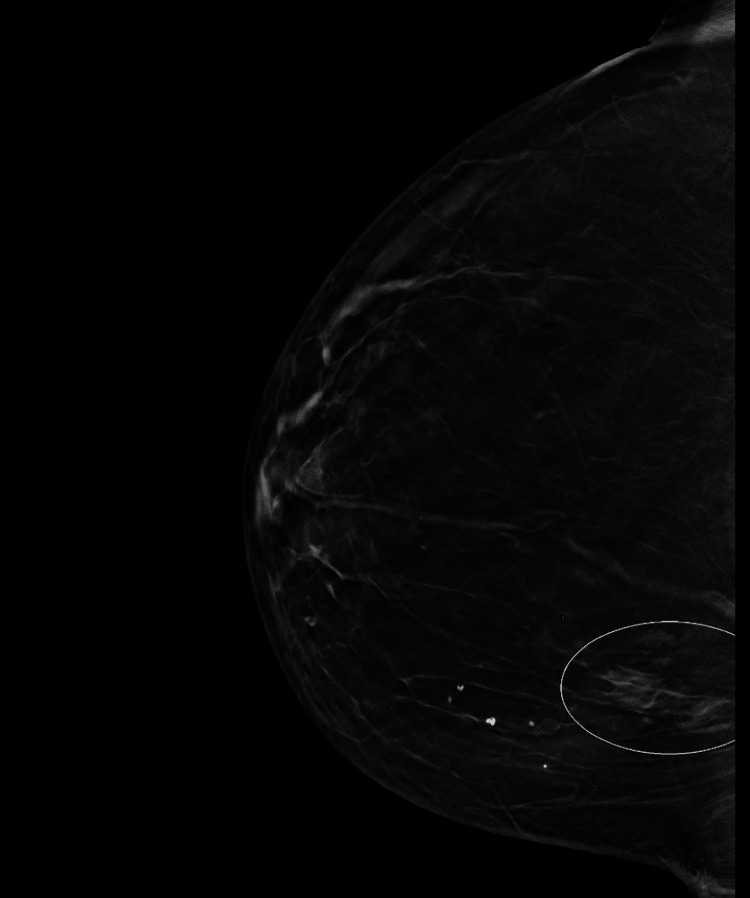
Right breast mammography Mammography of the right breast reveals a heterogeneous hyperechogenicity (circled) categorized as BI-RADS 4. BI-RADS: Breast Imaging Reporting and Data System

A 74-year-old female patient presented to her local physician for a screening mammogram. The patient was asymptomatic at the time; however, her past medical history was remarkable for undifferentiated connective tissue disease (UCTD) with inflammatory arthritis and features of Sjögren’s (Sicca) syndrome with borderline positive antinuclear antibody titers (1:40). She reported no personal history of cancer. No breast masses or axillary lymphadenopathy were discovered on physical examination. Mammography revealed no suspicious findings in the left breast. However, the right breast was remarkable for areas of heterogeneous hyperechogenicity in the posterior upper inner quadrant with a dominant area measuring 4.2 × 2.3 × 0.8 cm (Figure 1).

This area was interpreted as BI-RADS category 4, indicating moderate suspicion for malignancy. The patient underwent an ultrasound-guided core biopsy with clip placement without complications, and two tissue cores were sent to pathology for evaluation.

**Figure 2:**
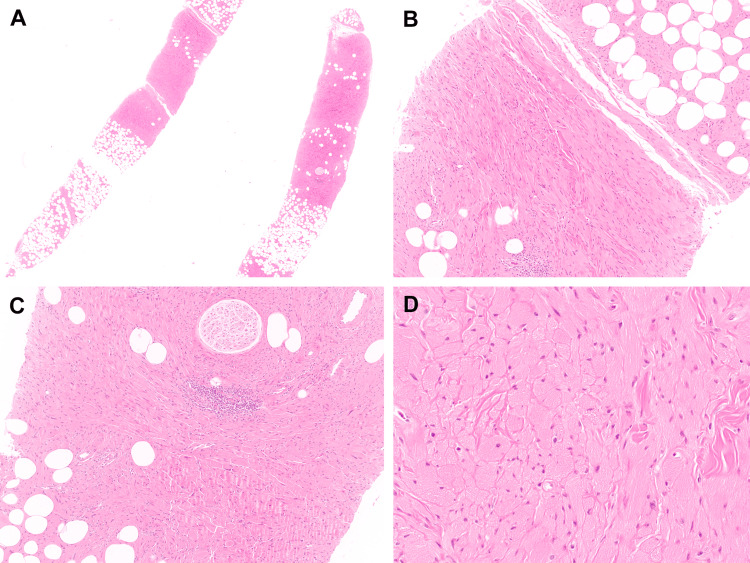
Crystal-storing histiocytosis of the breast A: At low power, the core biopsy fragments demonstrate a distinct eosinophilic cellular proliferation traversing adipose tissue without obvious native breast parenchyma present (H&E, 1×). B: The lesional cells have a vague fascicular-like pattern and involve the surrounding adipose tissue (H&E, 10×). C: A nerve and associated vessels are entrapped but uninvolved by the lesional proliferation. A small focal chronic lymphocytic inflammatory collection is noted (H&E, 10×). D: The lesional cells are haphazardly arranged in sheets with small, bland ovoid nuclei and abundant eosinophilic cytoplasm without nuclear atypia, mitotic activity, or necrosis (H&E, 20×). H&E: hematoxylin and eosin

Hematoxylin and eosin (H&E)-stained sections revealed two core biopsy tissue fragments with dense and diffuse sheets of polygonal to spindled plump histiocytes with abundant eosinophilic cytoplasm containing striated crystalline material (Figure 2).

**Figure 3:**
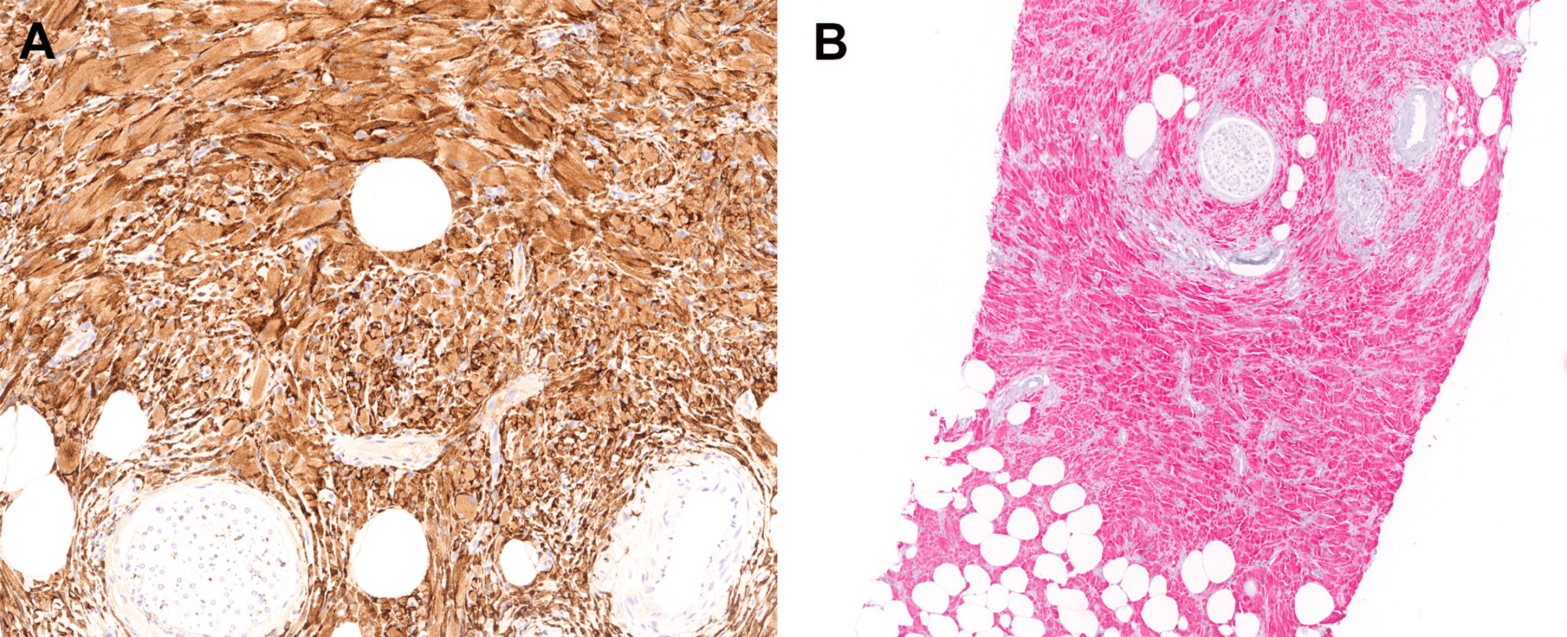
CD163 and NKI-C3 immunohistochemical stains A: Immunohistochemical stain for CD163 (DAB, 20×) is strongly and diffusely positive. B: Immunostain for NKI-C3 (red chromogen, 10×) demonstrates similar strong and diffuse positivity in the lesional cells. DAB: 3,3'-diaminobenzidine

The histiocytes were arranged haphazardly and in loose fascicles, with few small lymphocytic aggregates noted focally. Native breast epithelium was not identified. There was no evidence of increased mitotic activity, necrosis, or cytologic atypia. Given the lesional cells’ histiocytoid appearance, CD163 and NKI-C3 immunostains were performed. The lesional cells demonstrated diffuse cytoplasmic expression of CD163 and NKI-C3 by immunohistochemistry, consistent with an activated macrophage cell of origin (Figure 3).

**Figure 4:**
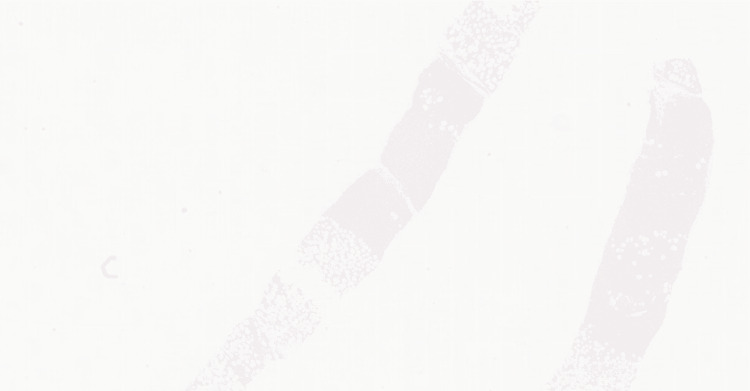
Representative image of the negative immunostains A representative image demonstrating negative staining of the lesional cells for S100, SOX10, HMB45, MelanA, cytokeratin AE1/AE3, desmin, caldesmon, myogenin, and MyoD1 immunostains (4× magnification).

Immunostains for S100, SOX10, HMB45, MelanA, cytokeratin AE1/AE3, desmin, caldesmon, myogenin, and MyoD1 were all negative (Figure 4).

Based on the morphologic findings and ancillary study results, a diagnosis of “crystal-storing histiocytosis” was rendered, with a comment issued informing the patient’s clinical provider that the patient should undergo evaluation for a potential lymphoproliferative disorder due to frequent etiologic association with CSH. It was also noted that the patient’s suspected autoimmune disorder may be contributory but is a less frequent etiologic factor in CSH.

A thorough workup was performed on the patient, and it was noted that the disease was limited only to the right breast at the time, with no other systemic manifestations. Further laboratory testing (Table 1) were ordered by the patient’s physician and revealed mild leukopenia (3.53k/mcL, reference range: 4.80-10.80 mcL) with mild absolute neutropenia (1.83k/mcL, reference range: 2.06-7.02k/mcL) and an elevated mean platelet volume (12.2 fL, reference range: 7.4-10.4 fL) on complete blood count. Bone marrow aspiration and biopsy were not performed. Antinuclear antibodies were identified at a titer of 1:40, indicating borderline positivity, without a clinically reportable degree of fluorescence. Reflex testing for beta-2 microglobulin antibodies was positive (2.50 mg/L, reference range: 1.00-2.00 mg/L), with elevations in beta-2 microglobulin IgG (91 GPI IgG units, reference range: 0-20 GPI IgG units). Antiphosphatidylserine, anticardiolipin, and cardiolipin antibodies were not elevated. The dilute Russell’s viper venom time (DVV) ratio was within normal limits (1.0, reference range: <1.3 ratio), negative for lupus inhibitor. Coagulation studies (activated partial thromboplastin time, prothrombin time, international normalized ratio, and thrombin time) were within normal limits. IgM levels were reduced (38 mg/dL, reference range: 63-277mg/dL), while IgG and IgA levels were within normal limits. No monoclonal proteins were detected by serum immunofixation, and kappa and lambda free light chains and ratio were within normal limits. Serum protein electrophoresis was additionally unremarkable, with no monoclonal proteins detected. Furthermore, no clonal T-cell receptor beta populations were detected by capillary electrophoresis. Follow-up mammography indicated no significant change in the lesion with appropriate biopsy clip placement and no indications for surgical intervention. Overall, the laboratory findings were clinically worrisome for antiphospholipid syndrome, type 4 (in combination with granulocytopenia). Definitive diagnosis of antiphospholipid syndrome could not be rendered due to the patient’s lack of historical thromboembolic disease or pregnancy morbidity; however, in a setting of high-risk laboratory findings, the patient was placed on daily low-dose aspirin for clot prophylaxis.

**Table 1:** Summary of laboratory results with references L*: below reference range, H*: above reference range WBC^a^: white blood cell, ANA^a^: antinuclear antibody, Ab: antibody, DVV^a^: dilute Russell’s viper venom time, PTT^a^: activated partial thromboplastin time, PT^a^: prothrombin time, INR^a^: international normalized ratio

Test	Result	Reference range
WBC^a^ count (k/mcL)	3.53 (L*)	4.5-11.0
Neutrophils (k/mcL)	1.83 (L)	2.06-7.02
Mean platelet volume (fL)	12.2 (H*)	7.4-10.4
ANA^a^	1:40 (H)	<1:40 (negative)
Beta-2 microglobulin (mg/L)	2.50 (H)	1.00-2.00
Beta-2 glycoprotein I Ab, IgG (GPI)	91 (H)	0-20
Antiphosphatidylserine Abs (U)	IgM: <10, IgA: <1, IgG: 10	IgM: 0-30, IgA: 0-19, IgG: 0-30
Anticardiolipin Abs (U/mL)	IgM: <9, IgG: <9	IgM: 0-12, IgG: 0-14
Cardiolipin (IgA) Ab (U/mL)	<9	0-11
DVV^a^ ratio	1.0	<1.3
aPTT^a^ (seconds)	28	24-33
PT^a^ (seconds)	10.9	9.1-11.7
INR^a^ (ratio)	1.0	<4.0
Serum immunofixation electrophoresis	No monoclonal proteins detected	No monoclonal proteins detected
Kappa free light chain (mg/dL)	2.11	0.82-2.89
Lambda free light chain	2.37	0.91-3.26
Kappa/lambda ratio	0.89	0.53-1.51
T-cell receptor beta clonality	Negative for clonality	Negative for clonality

## Discussion

CSH is a very uncommon associated finding in patients with lymphoproliferative disorders but a far rarer discovery in the absence of hematopoietic neoplasia. In a non-neoplastic setting, an autoimmune, inflammatory, infectious, environmental, or drug-related etiology may be explored. Patients with CSH are typically asymptomatic regarding the disease; however, CSH may serve as the first clue of a hematopoietic neoplasm, autoimmune disorder, or inflammatory process, and thus is imperative to detect in order to better enable diagnosis of a more urgent associated entity. Additionally, while most often identified after diagnosis of an associated condition, there are rare instances where CSH is the initially presenting pathology [3,4,7,9]. In this setting, pathologists play a vital role not only in the diagnosis of CSH but also in guiding clinicians toward the workup of an associated entity, with an emphasis on malignant associations. Electrophoretic testing for monoclonal gammopathy and laboratory investigation for autoimmune, inflammatory, or infectious etiologies are warranted in the absence of a pre-existing cause of CSH. In our case, detection of CSH in the breast urged the patient’s physician to order additional testing, ruling out clinical signs of a hematopoietic neoplasm and identifying laboratory indicators of possible antiphospholipid syndrome, resulting in prophylactic antithrombotic treatment.

Few reports of CSH involving breast tissue exist in the literature [4,15-19]. In all previously described cases (n = 6), an associated atypical or malignant hematopoietic process was identified in conjunction with CSH. These included associated extranodal marginal zone lymphoma in four cases (67%), low-grade B-cell lymphoma, not otherwise specified (17%), and an “atypical lymphoplasmacytic infiltrate” (17%). CSH was localized to the breast in four cases (67%), while accompanying involvement of the bone marrow, head and neck, and/or chest wall was observed in the two remaining cases (33%). The atypical hematopoietic process was identified by histopathology adjacent to or admixed with CSH in five of six cases (83%). To the best of our knowledge, our case represents the first patient with localized breast CSH in the absence of an associated lymphoproliferative disorder.

In the non-neoplastic setting, CSH is most often associated with Sjögren’s syndrome, followed by rheumatoid arthritis [7-11]. Our patient exhibited symptoms encompassing a variety of potential autoimmune etiologies, most notably symptoms of inflammatory arthritis and Sjögren’s syndrome, without definitive laboratory confirmation. The etiology of the patient’s CSH may be related to her reported symptoms, which align with the most common non-neoplastic associated etiologies of CSH. However, additional serologic workup after CSH diagnosis revealed elevated beta-2 microglobulin IgG antibodies and borderline positive antinuclear antibody titers. To the best of our knowledge, only a single case report demonstrates the co-occurrence of CSH and antiphospholipid syndrome [20]. The case, involving a 38-year-old female patient with known antiphospholipid syndrome and multiple lung nodules, revealed CSH in close proximity to a more closely associated entity, pulmonary extranodal marginal zone lymphoma. Therefore, the relationship between CSH and antiphospholipid syndrome is not yet established thus far.

Histologically, the abundant cytoplasm and striated crystals in CSH may resemble other entities, including granular cell tumor, melanocytic neoplasm, rhabdomyoma, and alveolar soft part sarcoma, all of which can be distinguished by immunohistochemical or molecular ancillary studies. In our case, the diffuse cytoplasmic expression of CD163 and NKI-C3, and negative staining for schwannian markers, S100/SOX10, or myogenic markers, desmin/caldesmon/myogenin/MyoD1, confirmed the histiocytic nature of the lesional cells and essentially excluded the diagnosis of granular cell tumor and rhabdomyoma. The negative melanocytic markers HMB45 and MelanA argued against a melanocytic neoplasm, and the negative FISH for TFE3 gene rearrangement basically ruled out an alveolar soft part sarcoma. Careful morphologic examination and a thorough immunohistochemical and molecular ancillary testing are important diagnostic tools in reaching the right diagnosis for this entity and avoiding diagnostic pitfalls.

## Conclusions

In summary, CSH is a rare entity that can arise in the context of both malignant and benign plasma cell proliferations or immune dysregulation, as well as, in rare instances, non-immunoglobulin-related etiologies. Herein, we present a case of CSH occurring in an unusual location in the absence of an associated hematopoietic malignancy, for which additional investigation into the patient’s undifferentiated connective tissue disease revealed positive serology for antiphospholipid antibodies. This case underscores the highly variable presentation of this condition, as well as its rare non-neoplastic associations. Further, it illustrates the key role of the pathologist in histopathological detection of CSH and the importance of evaluating for associated entities when diagnosed in isolation, as CSH may represent the first manifestation of the disease.
